# Structure-Mechanical Property Relations of Skin-Core Regions of Poly(p-phenylene terephthalamide) Single Fiber

**DOI:** 10.1038/s41598-018-37366-0

**Published:** 2019-01-24

**Authors:** Sakineh Chabi, Dmitriy A. Dikin, Jie Yin, Simona Percec, Fei Ren

**Affiliations:** 1 Department of Mechanical Engineering, Temple University, Philadelphia, Pennsylvania 19122 United States; 2 Department of Mechanical Engineering, University of New Mexico, Albuquerque, New Mexico 87131 United States; 3 College of Science and Technology, Temple University, Philadelphia, Pennsylvania 19122 United States

**Keywords:** Mechanical engineering, Mechanical properties

## Abstract

This study aims to elucidate the relationship between the mechanical properties and microstructures of poly(p-phenylene terephthalamide) (PPTA) single fibers at the micro/nano scale. The skin-core structure of Kevlar® 29 fiber was revealed through a focused electron beam experiment inside a scanning electron microscope (SEM) chamber. Cross sectional SEM images of the broken fiber showed that the thickness of the skin ranged from 300 to 800 nm and that the core region consisted of highly packed layers of fibrils. The skin and the core regions showed different mechanical behaviour and structural changes during nanoindentation and micro-tensile tests, indicating that the core region possessed higher stiffness, whereas the skin region could undergo more plastic deformation. Furthermore, micro-tensile testing results showed that the ultimate tensile strength, the elongation at failure, and the tensile toughness of single fibers could be significantly enhanced by cyclic loading. Such findings are important to understand the contribution of different microstructures of Kevlar® fibers to their mechanical performance, which in turn can be utilized to design high-performance fibers that are not limited by the trade-off between toughness and stiffness.

## Introduction

The use of poly(p-phenylene terephthalamide) (PPTA), such as DuPont’s Kevlar® fibers, in applications seeking strong and lightweight materials is well documented. The unrivalled mechanical behaviour of these fibers is undoubtedly a result of their microstructures. Using a variety of experimental techniques, researchers have uncovered distinct structural features of Kevlar® fibers^1–7^. The chemical structure of Kevlar® is presented in Fig. S1, in which the aromatic rings are linked to the backbone chain through the *para-* positions, and the adjacent chains bond together *via* hydrogen bonds to form pleated sheets. It is believed that the extended chain orientation derived from the linear geometry of the *para-* linkage is primarily responsible for the exceptional mechanical strength of Kevlar® fibers^3,8–10^. In addition to the extended chain, the morphological characteristics of the polymer significantly affect the mechanical properties and, as such, the deformation and fracture mechanisms of the polymer^3,11^.

To date, various models have been proposed to describe the microstructure of Kevlar® fibers including pleat sheet, fibrillary, and skin-core. The skin-core model has been verified and became a prevailing model describing the structure of Kevlar® fibers. The skin-core morphology is developed during the coagulation stage due to different coagulation rates between the outer and inner regions of the fiber. During the coagulation stage in the spinning process, polymer chains located near the surface are immediately aligned, while those in the centre are relaxed^1,3,12–14^. Consequently, the skin and the core portions of the fiber are different in terms of crystallinity, orientation level, void amount, and hence mechanical properties. While the core region has higher crystalline structure due to a slower cooling rate^1,12–14^, rapid formation of the skin results in a less crystalized but highly coherent structure as the chain ends do not have enough time to crystalize and form clusters. The amorphous nature of the skin has been confirmed by Dobb and co-authors^1^ and others^4,5,15^. It is important to mention that skin-core effects and morphology are strongly affected by the fiber type. Different Kevlar® fibers might have different skin thicknesses, different moisture, and microvoid levels, due to different post-coagulation treatments. Recently, focused ion beam, nanoindentation, and interfacial force microscopy have been used to study the effects of skin-core morphology in Kevlar® 49 and KM2 fibers^4,16–18^.

In this work, we aim to explore new aspects of the skin-core morphology of Kevlar® 29 fibers and specify their mechanical properties. To achieve this, we used a focused electron beam to reach the core of the fiber, and then we performed micro/nano mechanical tests to identify the role of each microstructural region. This work provides new insights into the structure-property relationship of Kevlar® 29 single fibers and paves the way for further improvement of high-performance polymer fibers.

## Results and Discussion

General information about the structure and crystallinity of Kevlar® single fibers was obtained by using SEM (scanning electron microscopy), EDS (energy dispersive x-ray spectroscopy) and XRD (x-ray diffraction) techniques, Fig. S2. Both optical and SEM micrographs showed that the average diameter of the single fiber is approximately 12 µm, and EDS analysis shows signals for C, O, N, and a small amount of S (possibly originating from the residual sulfuric acid trapped inside the fiber). A typical XRD profile of Kevlar® 29 fiber is shown in Fig. S2c, which indicates the fiber has both crystalline (intense peaks) and amorphous characteristics (broad peaks and a non-zero baseline). The two peaks at 2θ values of 20.5 and 22.8 degrees can be indexed to the (110) and the (200) crystallographic planes, respectively. It has been discussed that the crystalline portion of the fiber is related to the stacked sheets of hydrogen-bonded, fully extended polymer chains, and the amorphous region is due to less ordered structures, chain ends, and tie molecules^19^.

**Figure 1:**
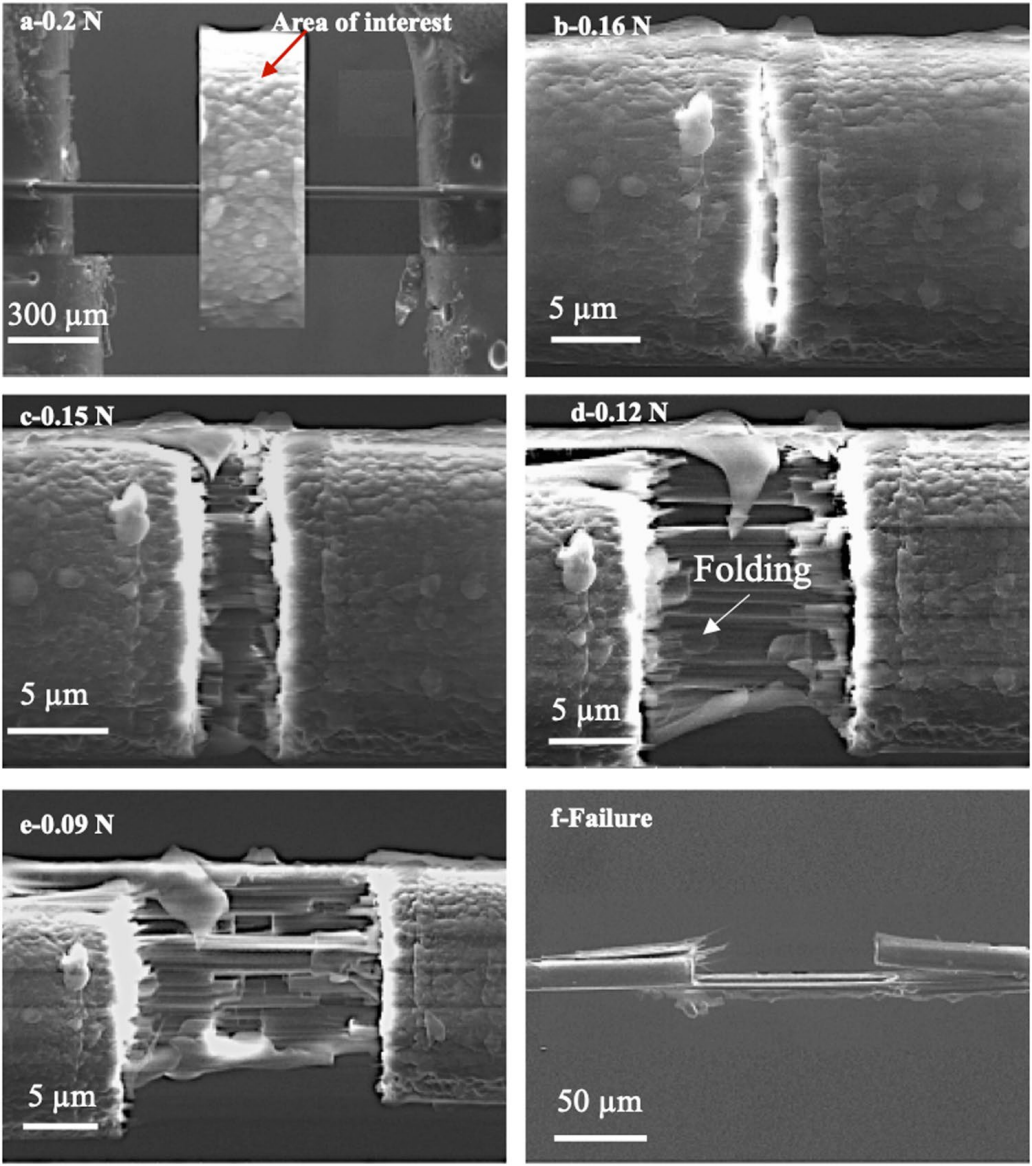
SEM images of Kevlar® 29 fiber during focused electron beam experiment: (**a**) prior to the experiment, (**b**–**e**) at different exposure time during the experiment, and (**f**) after the failure occurred.

A detailed investigation of the internal and external structures of the fiber was achieved by creating a sharp cut in the fiber by using a focused electron beam. Figure 1 presents a series of sequential SEM images of the fiber during the SEM experiment. Since the fiber was already stressed with a preload of 0.2 N, subjecting it to a focused electron beam bombardment weakened the polymer and resulted in ultimate failure. As shown in Fig. 1, the fiber exhibited a stepwise drop in the load as a result of consecutive fractures of fibril layers. As the scanning proceeded, the most stressed layers of the fiber, evidently the outer layers, failed first. Further, as seen in Figs. 1d&e, some fibrils showed a self-folding effect at the broken ends of the fibrils, which likely occurred to reach a stable energy level. It has been discussed that the polymer chain can only fold over itself in a crystalline region (as opposed to the skin)^20^.

**Figure 2:**
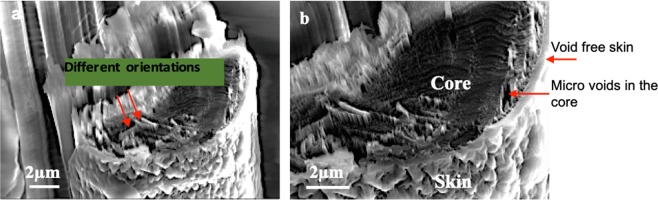
SEM images of cross sectional area of Kevlar® 29 fiber fractured in the focused electron beam experiment.

Cross-sectional SEM images of the broken fiber, Fig. 2, reveal the skin-core structure of the Kevlar® fiber, in which the thickness of the skin varies from 300 nm to 800 nm. The variation in the thickness of the skin could be related to the synthesis processes and parameters, such as the spinning rate. Unlike the core, which consists of radially oriented pleated sheets, the skin does not reveal any visible fibrous structure, Fig. 2. On the other hand, the skin seems to be a void-free, coherent region, wherein there are some micro-voids in the core.

As seen in Fig. 2b, these voids are located mainly between radially arranged pleated sheets. These voids are formed during the drying stage, where subsequent loss of solvent molecules from the core region results in the voiding of the interior structure^1,21^. Coherent appearance of the skin might be due to the existence of moisture in this region. Thus, the skin and the core have completely different appearances, morphologies, and microstructures.

This being understood, the following questions naturally arise: How do these two regions contribute to the mechanical performance of the fiber? How do they behave during failure? Which one is stiffer and stronger? To answer these questions, the mechanical properties of Kevlar® 29 single fibers were measured at both the micro and nano levels, and the relationship between these properties was investigated for both the skin and the core regions of the fiber.

### Micromechanical testing

**Figure 3:**
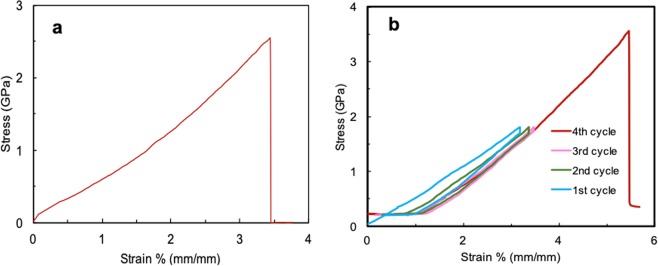
Typical stress-strain curves during a tensile test (**a**), and a tensile cyclic test (**b**) of Kevlar® 29 fibers.

To reveal the microscopic mechanical properties of the fiber, we used a micro-tensile test to characterize the stress-strain curves of the fiber and the results are shown in Fig. 3. As seen in Fig. 3a, the stress-strain curve of Kevlar® 29 fiber consists of three different regions: the initial nonlinear region (0–1.5% strain), which is known as the toe region^22^; the linear region (1.5–3.4% strain); and the failure region (at 3.4% strain). The toe region corresponds to the removal of crimps from the fibrils. The tensile strength of the fiber is approximately 2.5 GPa, and the fiber breaks at a strain level of 3.4%, which agrees well with the data reported in the literature^23^. The tensile toughness of the fiber, which is equal to the area under the stress-strain curves, was found to be 4.2 GJ/m^3^. The initial slope of the curve corresponds to a Young’s modulus of approximately 75 GPa, and the slope increases at a higher strain level (>1.5%) and gives a modulus of approximately 80 GPa in the linear region. The increase in modulus is a result of better alignment of the polymer chains at higher strains, which agrees with the fact that the extended chain orientation is primarily responsible for the superior mechanical behaviour of Kevlar® polymers.

The micromechanical properties of Kevlar® single fiber was further explored under cyclic tensile loading and unloading conditions, Figs 3b and S3. A tensile load of approximately 75% (in Fig. 3b) and 60% (in Fig. S3) of the fracture force was applied to conduct the cyclic tensile testing (cycles 1–3). As seen from Fig. 3b, the shape of the strain-stress curves is affected by the number of cycles. As the number of cycle increases, from 1 to 3, the hysteresis in the stress-strain curves, corresponding to the energy dissipation in the fiber, shrinks and almost disappears in the 3^rd^ cycle, suggesting the elimination of the viscous component and full domination of the elastic behaviour. Such a phenomenon was reported elsewhere as well^24–26^. Further, subjecting the polymer to the cyclic loading leads to the evolution of residual strain. However, the amount of residual strain decreases with the increase in cycle number until it reaches a saturation level in the 3^rd^ cycle, where no further residual strain remains.

Interestingly, when subjected to cyclic tests (Fig. 3b), the fiber shows much higher tensile strength (3.6 ± 0.2 GPa vs. 2.6 ± 0.2 GPa) and elongation at break, and thus higher tensile toughness (9 GJ/m^3^) than the as-received fiber, shown in Fig. 3a. We believe that the increased toughness is related to the microstructural changes during cyclic loading that contribute to the increased resistance to major crack growth. This may be generated via mechanisms such as crack deflection, microcracking, crack shielding, and crack bridging^27–30^. For example, in the case of Kevlar fiber®, some fibrils can act as bridges to inhibit crack propagation or change the path in which a crack propagates in the fiber^28^.

**Figure 4:**
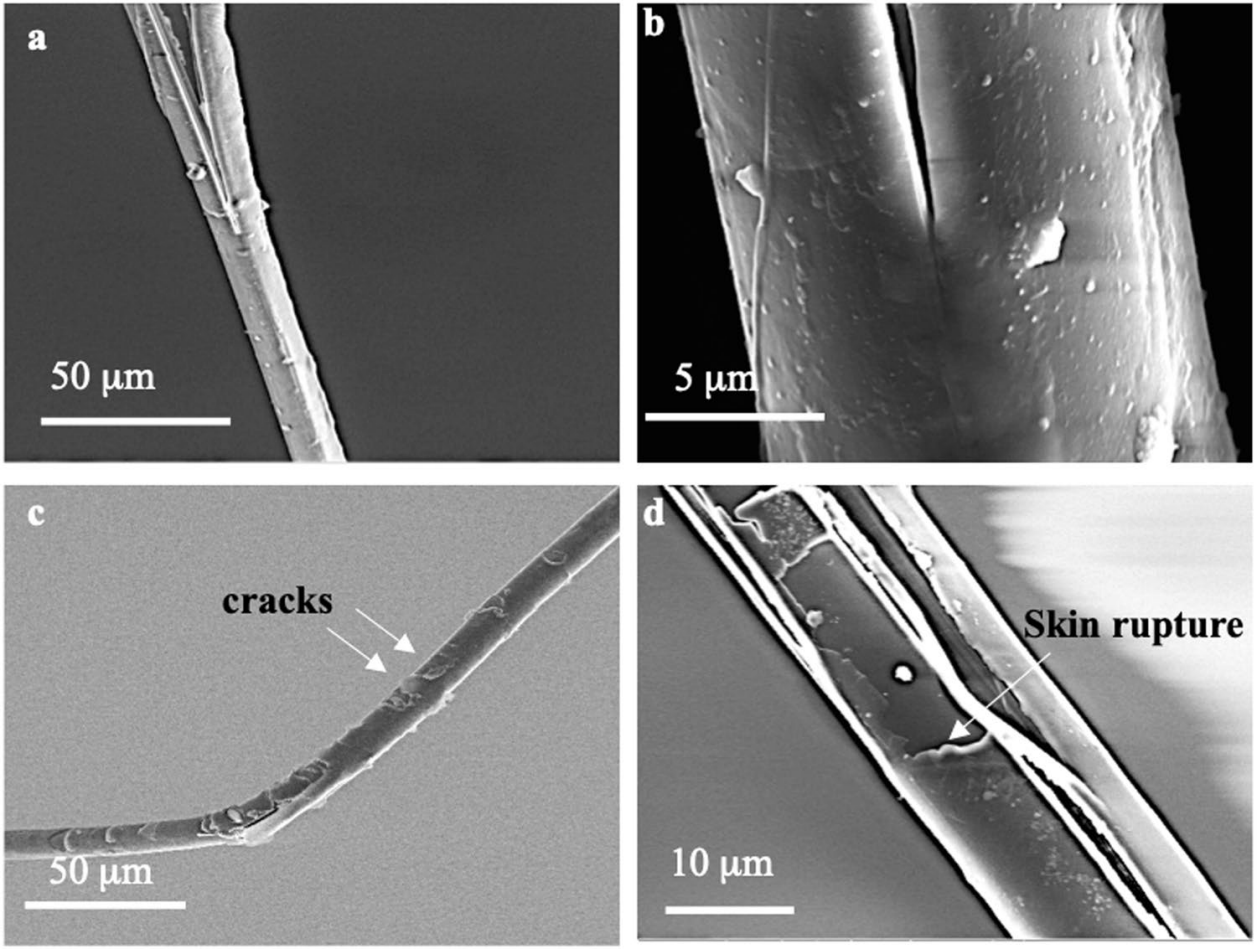
SEM images of fracture surfaces of (**a**,**b**) the as-received fiber and (**c**,**d**) the cyclically loaded fiber, where cracks can be seen on the fiber surface.

Fracture regions of the as-received fiber and the cyclically loaded fiber are compared in Fig. 4. Although, both fibers fail via fibrillation and pull-out modes, there is significant difference in the surface of the fibers. In the as-received fiber, shown in Figs. 4a&b, there are no microcracks on the surface of the fiber, and there is only one major crack path in the fiber which is alongside the fiber axis. It is even difficult to distinguish between the skin and core, as the fiber looks like a single unit. However, in the cyclically loaded fiber, there are many cracks on the surface, and skin rupture occurs here, as shown in Figs. 4c&d. These cracks, appearing in different directions and forms, may help in both releasing the stress locally and changing the crack path. Further, given that the main interface here is the skin-core interface, and given the fact that skin rupture takes place here, it is likely that the residual strain and the subsequent crack evolution evolves at the skin/core interface. In fact, due to the structural inhomogeneity in the fiber (e.g., between the skin and core), phase separation occurs upon cyclic loading that eventually generates extrinsic toughness in the polymer^30,31^. Moisture-induced plastic toughening has been reported for other fibers^32^. Whether the toughening behavior observed in the current study is affected by the moisture content of the skin demands further investigation in the future. Further, depending on the fiber diameter, the skin and core might contribute differently to the mechanical properties of the fiber. The effects of the fiber diameter on the mechanical properties of the core and skin had been reported for protein fibers as well^33^.

### Nanoindentation testing

**Figure 5:**
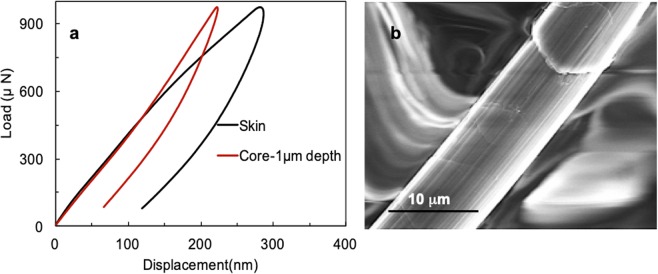
(**a**) Load-displacement results of the skin and core regions. The penetration depth for the skin and core measurement is about 250 nm and 1μm respectively. (**b**) SEM image of a Kevlar® 29 fiber without skin (i.e. skinless fiber).

Having characterized micro-tensile behaviour of the polymer, we then used nanoindentation technique to measure the local mechanical properties of the fiber at the nanoscale. Figure 5a presents typical load-displacement curves from nanoindentation measurement in two different regions: the skin and the core. For the skin measurement, the indentation test was performed on the skin region of the fiber; for the core measurement the indentation test was performed at a depth of 1 µm (which is in the core region). Both experiments were carried out in load control mode (peak force of 1000 µN). As seen in Fig. 5a, the area under the load-displacement curve of the skin is much larger than that of the core, indicating that the skin has a larger energy dissipation capability than the core; thus, the skin indeed plays a profound role in the toughness of the polymer.

The stiffness of each region can be investigated from the slope of the unloading part of the curves. As seen in Fig. 5a, the load-displacement curve of the core has higher slope than that of the skin; thus, the core is stiffer than the skin. We obtained values of approximately 9.5 and 6.65 GPa as the reduced modulus, E_r,_ for the core and skin, respectively. Then, using the equation E = 0.87 E_r_ (see Supporting Information), values of 8.2 and 5.6 GPa were obtained as the elastic moduli of the core and skin, respectively. The hardness, H, of the two regions was also measured by nanoindentation experiments, and values of 1.8 and 0.9 GPa were obtained for the core and skin, respectively. These results agree well with our earlier observations and with previous studies, and the difference in the moduli is related to the difference in the microstructures and intermolecular bonding in the two regions. The higher stiffness of the core is a result of increased crystallinity of this region^1,14,16,17^.

To further visualize the structural differences between the skin and the core, we then used a chemical approach to exfoliate the skin and prepare fibers without skin, as shown in Fig. 5b. In fact, Fig. 5b clearly shows that the intermolecular bonding in the core is much stronger than that in the skin, because the skin did not withstand the chemical etching as did the core. The average diameter of all chemically etched fibers was 10 μm, implying that the core benefits from much stronger intermolecular bonds and a denser packing than the skin. These findings agree well with the amorphous/crystalline description used earlier for skin-core regions. In addition, the findings suggest that depending on the application, specific structural engineering of the fiber or fabrics might be required. For example, materials for body armour require both stiffness and toughness; however, one of the main drawbacks of high-performance polymers such as Kevlar® is their low toughness. However, we now realize that this challenge might be addressed by controlling the structure of the skin during the processing (e.g., the thickness or roughness level of the skin), as the skin likely formed during the spinning process.

## Conclusions

In this work, we used a combination of microstructural and mechanical characterization techniques to differentiate the morphology and mechanical properties between the skin and core regions of Kevlar® 29 fiber. The major difference between the two regions is that the core possesses a fibrous structure and is stiffer with higher elastic modulus than the skin. On the other hand, the skin has an amorphous structure that imparts partial plasticity to the polymer, which is required for high toughness. Our findings indicate that the superior mechanical behaviour of Kevlar® 29 polymer comes from both the skin and core segments. These two regions contribute differently but cooperatively to the ultimate performance of the polymer. It is expected that adjusting processing and microstructure, such as the cooling conditions and thickness of the skin, could lead to further optimization of high performance fibers. Furthermore, the mechanical testing strategy used in this work may be used to investigate other types of fibers as well, e.g. biological fibers, where compositional or structural gradients exist.

## Methods

### Structural characterization

For the sample preparation, single microfibers were taken and cut from a bundle of Kevlar® 29 fibers. DuPont™ Kevlar 29® fibers were purchased from Goodfellow Corp. (Coraopolis, PA, USA). The morphology and structure of the fiber including fiber diameters and elemental composition were characterized using an FEI QUANTA 450FEG scanning electron microscope (SEM) with energy dispersive X-ray spectroscopy (EDS). The crystal structure of the fiber was investigated by using X-ray diffractions. XRD profiles were recorded using a Bruker D8 Advance diffractometer working with a Cu-Kα radiation (*λ* = 0.154 nm) operated at 40 kV and 40 mA. Samples for the XRD tests were prepared by wrapping the fibers around a glass slide.

### Focused electron beam experiment

Detailed information about the fiber microstructure was obtained by performing focused electron beam experiments. In this process, the fiber was subjected to both mechanical load and energetic electrons inside the SEM. In a typical test, a single Kevlar® fiber was clamped between the jaws of a micro-tensile testing stage (Deben Ltd., UK), as shown in Fig. S4a, and an axial preload of 0.2 N was first applied on the fiber. Then, a focused electron beam was set to scan along one line across the stressed fiber until a surface crack was initiated. The scanning process was repeated few times until the entire fiber failed. The SEM was operated at an acceleration voltage of 10 kV in a low vacuum mode with a pressure of 40 Pa.

### Mechanical characterizations

Micro and nano mechanical characterizations of the fiber were conducted by using the aforementioned micro-tensile testing stage and nanoindentation (TI980 TriboIndenter, Hysitron, Minneapolis, MN, USA). Fig. S4 shows the experimental setup for both tests. For micro-tensile testing, the fiber (20 mm in length) was placed on a specially designed paper sample holder, and then the two ends of the fiber were fixed by using epoxy resin. Tensile measurements were performed at the displacement rate of 0.1 mm/min.

For nanoindentation tests, the fiber (~30 mm long) was wrapped around a glass vial, and the two ends of the fiber were fixed on the vial using epoxy resin. Nanoindentation measurements were carried out with a diamond Berkovich indenter tip. All nanoindentation tests were conducted in the quasi-static domain. All indents were located along the fiber axis to avoid potential rotation of the fibers during test. To test the skin region, indentation depth was limited to 200–250 nm in order to minimize the substrate effect from the core.

### Preparation of skinless Kevlar® 29 fibers

Skinless Kevlar® fibers were prepared by etching the surface of the fiber by using a chemical approach. We slightly modified a previously reported procedure^7^. In this process, 0.1 g of Kevlar® 29 fibers was immersed into a 50 ml DMSO solution containing 0.1 g KOH, and then the solution was magnetically stirred for about one week at room temperature. This procedure resulted in the exfoliation of the skin from the core. The resulting fibers were rinsed several times in deionized water prior to SEM characterization. The average diameter of the skinless fibers was 10 μm.

## Supplementary information

Supplementary Information
