# Author Correction: Structure-Mechanical Property Relations of Skin-Core Regions of Poly(p-phenylene terephthalamide) Single Fiber

**DOI:** 10.1038/s41598-019-43509-8

**Published:** 2019-05-03

**Authors:** Sakineh Chabi, Dmitriy A. Dikin, Jie Yin, Simona Percec, Fei Ren

**Affiliations:** 1 Department of Mechanical Engineering, Temple University, Philadelphia, Pennsylvania 19122 United States; 2 Department of Mechanical Engineering, University of New Mexico, Albuquerque, New Mexico 87131 United States; 3 College of Science and Technology, Temple University, Philadelphia, Pennsylvania 19122 United States

Correction to: *Scientific Reports* 10.1038/s41598-018-37366-0, published online 24 January 2019.

In the Supplementary Information file originally published with this Article, Supplementary Figures S1-S4 were omitted. These errors have been corrected in the Supplementary Information that now accompanies the Article.

