# Supplementary material for: Structure-Mechanical Property Relations of Skin-Core Regions of Poly(p-phenylene terephthalamide) Single Fiber
Source: Sci Rep. 2019 Jan 24;9:740. doi: 10.1038/s41598-018-37366-0 (PMC6345797; doi:10.1038/s41598-018-37366-0)
Supplement: Supplementary file 1 — Supplementary Information [file 41598_2018_37366_MOESM1_ESM.pdf]

Supporting Information:

Structure-Mechanical Property Relations of Skin-Core Regions of Poly(p-phenylene terephthalamide) Single Fiber

Sakineh Chabi[a,b], Dmitriy A. Dikin[a], Jie Yin[a], Simona Percec[c], and Fei Ren [a]*

[a]Department of Mechanical Engineering, Temple University, Philadelphia, Pennsylvania 19122, United States

[b] Department of Mechanical Engineering, University of New Mexico, Albuquerque, New Mexico 87131, United States

[b]College of Science and Technology, Temple University, Philadelphia, Pennsylvania 19122, United States

*Email: renfei@temple.edu

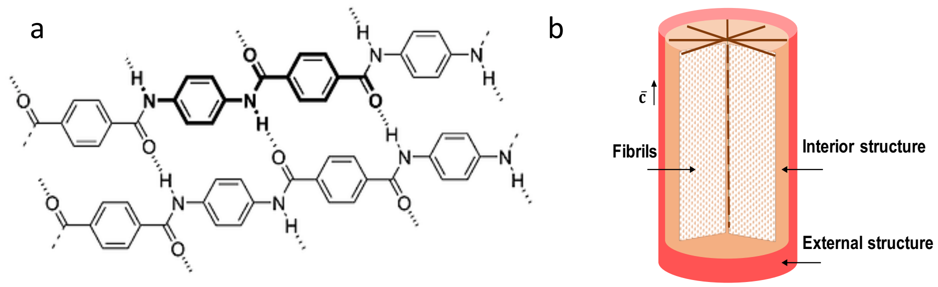

Fig. S1 Chemical structure (a) and a structural model (b) of PPTA.

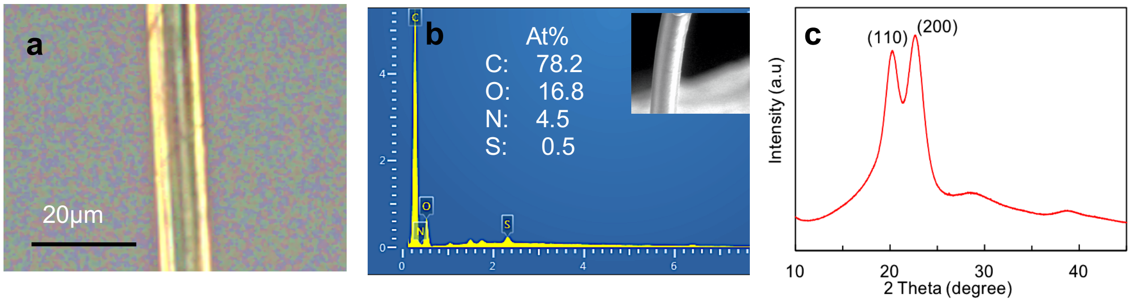

Fig .S2 Optical micrograph(a), EDX, SEM (b) and XRD spectrum (c) of Kevlar® 29.

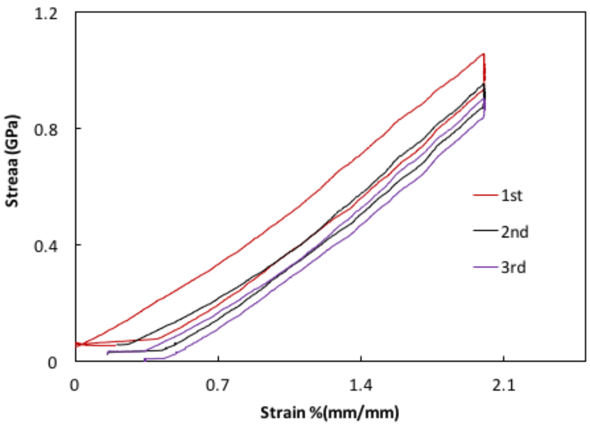

Fig. S3 Cyclic stress-strain curve of Kevlar®29 fiber. The hysteresis loss, which is proportional to the area of stress-strain curve, is much larger in the first cycle than that in the 2nd and 3rd cycles.

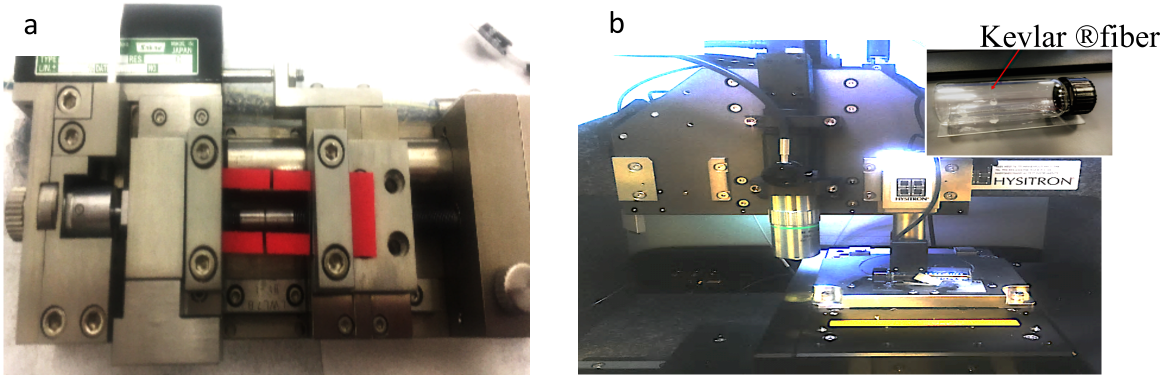

Fig .S4 A photograph of the micro tensile testing stage (a). Kevlar® single fiber (length 20mm) was mounted on a paper frame using an epoxy resin. A photograph of the nanoindentation testing stage during tests. As shown in the inset, Kevlar® fiber is wrapped around a glass vial (b) .

Calculation of Elastic modulus (E):

The elastic modulus of the fiber was calculated using the following equation:

$$\frac{1}{E_R} = (\frac{1-\nu^2}{E})_{Sample} + (\frac{1-\nu^2}{E})_{indenter}$$

Where Er is the reduced modulus, E is the elastic modules, and ν is the poison's ratio. For diamond indentor E indenter is 1140 GPa, and ν indenter is 0.07.[1]

By using poison's ration of 0.36 for Kevlar®29,[2] we obtained the following relationship between reduced modulus and elastic modulus.

$E_{Kevlar®} = 0.87 E_r$

References:

1. Hysitron. *Probe Calibration*. **33,** (2014).
2. Du Pont. *DuPont Kevlar Technical Guide*. *Kevlar Aramid Fibers* (2016).
